# Second round results from the Manchester ‘Lung Health Check’ community-based targeted lung cancer screening pilot

**DOI:** 10.1136/thoraxjnl-2018-212547

**Published:** 2018-11-12

**Authors:** Phil A Crosbie, Haval Balata, Matthew Evison, Melanie Atack, Val Bayliss-Brideaux, Denis Colligan, Rebecca Duerden, Josephine Eaglesfield, Timothy Edwards, Peter Elton, Julie Foster, Melanie Greaves, Graham Hayler, Coral Higgins, John Howells, Klaus Irion, Devinda Karunaratne, Jodie Kelly, Zoe King, Judith Lyons, Sarah Manson, Stuart Mellor, Donna Miller, Amanda Myerscough, Tom Newton, Michelle O’Leary, Rachel Pearson, Julie Pickford, Richard Sawyer, Nick J Screaton, Anna Sharman, Maggi Simmons, Elaine Smith, Ben Taylor, Sarah Taylor, Anna Walsham, Angela Watts, James Whittaker, Laura Yarnell, Anthony Threlfall, Phil V Barber, Janet Tonge, Richard Booton

**Affiliations:** 1 Manchester Thoracic Oncology Centre, Wythenshawe Hospital, Manchester University NHS Foundation Trust, Manchester, UK; 2 Division of Molecular and Clinical Cancer Sciences, Faculty of Biology, Medicine and Health, University of Manchester, Manchester, UK; 3 Manchester Clinical Commissioning Group, Macmillan Cancer Improvement Partnership, Manchester, UK; 4 Manchester Health and Care Commissioning, Manchester, UK; 5 Greater Manchester, Lancashire, South Cumbria Strategic Clinical Network, Manchester, UK; 6 Manchester City Council, Manchester, UK; 7 Department of Radiology, Royal Preston Hospital, Preston, UK; 8 Department of Radiology, Manchester Royal Infirmary, Manchester University NHS Foundation Trust, Manchester, UK; 9 Department of Radiology, Royal Blackburn Hospital, Blackburn, UK; 10 The Black Health Agency, Manchester, UK; 11 Macmillan Cancer Support, Manchester, UK; 12 Department of Radiology, Papworth Hospital, Cambridge, UK; 13 Department of Radiology, Christie NHS Foundation Trust, Manchester, UK; 14 Department of Radiology, Salford Royal NHS Foundation Trust, Salford, UK; 15 Department of Radiology, Stockport NHS Foundation Trust, Stockport, UK

**Keywords:** lung cancer

## Abstract

We report results from the second annual screening round (T1) of Manchester’s ‘Lung Health Check’ pilot of community-based lung cancer screening in deprived areas (undertaken June to August 2017). Screening adherence was 90% (n=1194/1323): 92% of CT scans were classified negative, 6% indeterminate and 2.5% positive; there were no interval cancers. Lung cancer incidence was 1.6% (n=19), 79% stage I, treatments included surgery (42%, n=9), stereotactic ablative radiotherapy (26%, n=5) and radical radiotherapy (5%, n=1). False-positive rate was 34.5% (n=10/29), representing 0.8% of T1 participants (n=10/1194). Targeted community-based lung cancer screening promotes high screening adherence and detects high rates of early stage lung cancer.

## Introduction

The National Lung Screening Trial (NLST) demonstrated a 20% reduction in lung cancer–specific mortality with annual low-dose CT (LDCT) screening of high-risk ever smokers compared with chest X-ray.^1^ A key requirement for screening implementation is to ensure services are accessible to those at greatest risk. In Manchester, we developed a community-based ‘Lung Health Check’ (LHC) approach to target high-risk smokers in deprived areas. LHCs were nurse-led and included calculation of lung cancer risk using the PLCO_M2012_ risk model. Those at higher risk were eligible for annual LDCT screening over two screening rounds. There was a high prevalence of lung cancer detection at baseline (T0; undertaken June to August 2016) (3%); most cancers were early stage (80%) and therefore radically treatable.^2^ Here, we report the results of the second screening round (T1; undertaken June to August 2017).

## Methods

A description of the screening pilot has previously been published.^2^ In brief, ever smokers aged 55–74 at participating general practices (n=14) were invited to a LHC; this consisted of 6-year lung cancer risk calculation (PLCO_M2012_),^3^ symptom assessment, smoking cessation advice and spirometry. Individuals at higher risk (defined as ≥1.51% over 6 years) were offered annual LDCT screening. All LDCT scans (Optima 660; GE Healthcare) were reported by National Health Service (NHS) consultant radiologists with an interest in thoracic radiology and classified as either negative, indeterminate or positive. Pulmonary nodules were managed in accordance with British Thoracic Society (BTS) guidelines adapted for an annual screening programme.^4^ Indeterminate scans required surveillance imaging at 3 months and positive scans had findings concerning for lung cancer requiring immediate assessment in the rapid access lung cancer clinic based in a specialist centre. A false positive was any screened individual referred to the lung cancer clinic who was not diagnosed with lung cancer. An interval cancer was defined as any lung cancer diagnosed outside of screening before the second-round scan (T1). Volume doubling times (VDTs) were calculated in accordance with BTS guidelines.^4^ VDT was estimated in those without a nodule at baseline (T0) by assuming the nodule appeared the day after the CT scan was performed and measured 1 mm. Lung cancers were managed in accordance with national guidelines.^5^ The seventh edition of TNM lung cancer staging manual was used.^6^ In this paper, the first screening round is referred to as T0 and the second screening round 12 months later as T1. Individuals with an indeterminate scan at T1 had a further LDCT scan 3 months later, which we refer to as the ‘3-month surveillance’ scan.

## Results

**Figure 1:**
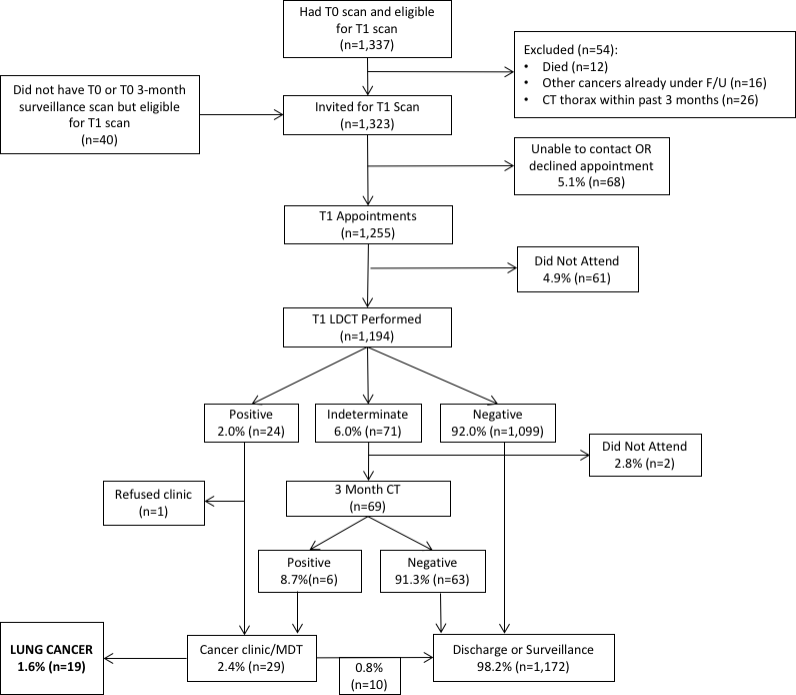
Diagram showing flow of participants through the screening service. LDCT, low-dose CT scan; MDT, multidisciplinary team.

Ninety per cent of those eligible had a T1 scan (June to August 2017) (n=1194/1323). Non-attendees were significantly more likely to be current smokers (63.6% vs 50.6%, p=0.005), but there was no difference according to deprivation (p=0.79) (table 1). The majority of T1 scans were ‘negative’ (92%, n=1099) (figure 1); 71 were ‘indeterminate’ of which 84.1% (n=58/71) were for nodule surveillance. The 3-month surveillance imaging rate was significantly lower than T0 (6% vs 13.7%; p=0.0001); six individuals were reclassified positive after 3-month scans. Overall, 30 scans were ‘positive’ (2.5%, n=30/1194)—one patient declined assessment. Of 29 individuals seen, 19 were diagnosed with lung cancer and 10 were not. The false-positive rate was 34.5% (n=10/29), which represents 0.8% of T1 participants (n=10/1,194). This false-positive rate was significantly lower (p=0.0001) than T0 (corresponding values 48.1% and 2.8%) and over both screening rounds it was 44.5% and 3.5%, respectively. There were no interval cancers between T0 and T1.

**Table 1:** Comparison of attendees and non-attendees of the second (T1) screening round

Variable		T1 Screening round		P values
		Attendees	Non- attendees	
No of attendees (%)		1194	129	–
Mean age (years±SD)		64.7 (5.4)	64.2 (5.6)	0.34
Sex M/F (F%)		587/607 (50.8)	65/64 (49.6)	0.79
Median IMD rank (IQR)		2848 (3615)	2908 (4195)	0.79
BMI (±SD)		28.5 (5.4)	28.3 (5.7)	0.73
Lung cancer risk (PLCO_M2012_±SD)		4.8 (3.8)	5.4 (4.7)	0.13
Education (%)	Less than ‘O’ level	822 (68.8)	93 (72.1)	0.58
	‘O’ level	213 (17.8)	24 (18.6)	
	‘A’ level	44 (3.7)	3 (2.3)	
	University/college	77 (6.4)	5 (3.9)	
	University degree	26 (2.2)	4 (3.1)	
	Postgraduate/professional	12 (1.0)	0	
Smoking status (%)	Current	604 (50.6)	82 (63.6)	0.005
	Former	590 (49.4)	47 (36.4)	
Smoking exposure (mean±SD)	Duration (years)	43.4 (8.3)	45.4 (7.0)	0.008
	Cigarettes/day	24.1 (12.8)	23.9 (12.5)	0.83
	Pack-years	51.2 (25.9)	53.4 (28.6)	0.37
Spirometry (mean±SD)	FEV_1_	2.16 (0.7)	2.08 (0.7)	0.26
	% predicted FEV_1_	84.9 (24.5)	81.0 (21.6)	0.09
	FVC	3.17 (1.0)	3.10 (1.0)	0.44
	% predicted FVC	100.4 (24.6)	96.3 (23.7)	0.07
	FEV_1_:FVC ratio	67.9 (10.7)	67.6 (12.3)	0.75
Airflow obstruction	Yes (%)	588 (49.6)	63 (53.1)	0.45
COPD/emphysema	Yes (%)	386 (32.2)	37 (28.7)	0.40
FH lung cancer	Yes (%)	326 (27.3)	32 (24.8)	0.54
MRC Dyspnoea Score (%)	1	781 (65.4)	72 (55.8)	0.13
	2	261 (21.9)	32 (24.8)	
	3	98 (8.2)	14 (10.9)	
	4	53 (4.4)	11 (8.5)	
	5	1 (0.1)	0	
Performance status (%)	0	655 (54.9)	60 (46.5)	0.12
	1	403 (33.8)	46 (35.7)	
	2	116 (9.7)	19 (14.7)	
	3	20 (1.7)	4 (3.1)	
	4	0	0	

BMI, Body Mass Index; FH, family history; IMD, Index of Multiple Deprivation; MRC, Medical Research Council.

The incidence of lung cancer in T1 was 1.6% (n=19/1,194), 79% were stage I (n=15), 10.5% stage III (n=2) and 10.5% stage IV (n=2) (table 2). Pathological subtypes included adenocarcinoma (32%, n=6), squamous cell (21%, n=4), small cell (16%, n=3) and non-small cell lung cancer not otherwise specified (10.5%, n=2). A clinical diagnosis was confirmed by the multidisciplinary team in four cases without pathological confirmation (21%). Cancer treatments included surgery (42%, n=9), stereotactic ablative radiotherapy (26%, n=5) and radical radiotherapy (n=1) (table 2). One individual had surgery for a benign lesion (granulomatous disease). There were no deaths within 90 days of surgery.

**Table 2:** Clinical details of screen detected lung cancers

T0 outcome	Stage	VDT (days)	Final stage	Pathology (subtype)	Treatment
Indeterminate	pT1a N0	369	IA	Adenocarcinoma (acinar)	Surgery
Indeterminate	pT1a N0	148	IA	Adenocarcinoma (acinar)	Surgery
Indeterminate	pT1a N0	89	IA	Squamous	Surgery
Indeterminate	pT1a N0	687*	IA	Adenocarcinoma (acinar 50%, solid 20%, lepidic 30%)	Surgery
Indeterminate	pT1a N0	206	IA	Squamous	Surgery
Indeterminate	pT1a N0	285	IA	Adenocarcinoma (micropapillary 50%, papillary 10%, lepidic 40%)	Surgery
Negative†	pT1a N0	142	IA	Adenocarcinoma (solid 80%, acinar 20%)	Surgery
Negative	cT1a N0	29‡	IA	Clinical	SABR
Negative†	cT1a N0	163	IA	Clinical	SABR
Negative†	cT1a N0	51	IA	NSCLC (NOS)	SABR
Negative	cT1a N0	71§	IA	Squamous	SABR
Negative	cT1a N0	67§	IA	Clinical	No treatment¶
Negative†	cT1b N0	65	IA	Clinical	Radical radiotherapy
Negative	pT2a N0	–	IB	Adenocarcinoma (solid 80%, lepidic 20%)	Surgery
Negative†	cT2a N0	72	IB	NSCLC (NOS)	SABR
Negative	cT1a N2	37‡	IIIA	Squamous	Chemoradiotherapy^(S)^
Negative	pT1a N2	86§	IIIA	Small cell	Surgery/chemotherapy^(A)^
Negative	cT4 N2 M1a	34‡	IV	Small cell	Chemoradiotherapy^(S)^
Negative	cT3 N3 M1b	16‡	IV	Small cell	Chemoradiotherapy^(S)^

*Morphology of nodule changed with increasing density despite low VDT.

†False negative, ^(S)^sequential treatment, ^(A)^adjuvant chemotherapy.

‡Estimated VDT.

§VDT calculated between T1 and T1+3-month surveillance scans.

¶Had chemoradiotherapy for oesophageal cancer.

NOS, not otherwise specified; NSCLC, non-small cell lung cancer; SABR, stereotactic ablative radiotherapy; VDT, volume doubling time.

Thirteen individuals with a negative baseline scan (T0) were diagnosed with lung cancer in the second round; after retrospective review, five were visible at baseline as sub-5 mm nodules and all were stage I at diagnosis (table 2). The T0 false-negative rate was therefore 0.4% (n=5/1337), negative predictive value 99.6%, sensitivity 89.4% and specificity 97.1%. The benign surgical resection rate over both rounds was 2.5% (n=1/40). Tumour VDT was highest in those with a true negative baseline scan (average 49±26 days), followed by false-negative (99±50 days) and indeterminate scans (297±215 days; p=0.009) (table 2).

## Discussion

In this paper, we report results from the second round of the Manchester ‘Lung Health Check’ pilot, a targeted lung cancer screening service based in deprived areas of Manchester. Screening adherence was high (90%) despite most participants being from the lowest decile of deprivation in England, emphasising the benefit of accessible community-based services. The incidence of lung cancer was 1.6% (n=19), most cancers were stage I (79%) and 89% of individuals with screen detected cancer were offered curative-intent treatment. Over both screening rounds, 4.4% of the cohort were diagnosed with lung cancer, equivalent to one cancer detected for every 23 people screened. This is high when compared with other studies and more than 2.5 times that seen in NLST (T0: 1.0%, T1: 0.7%) and NELSON (T0: 0.9%, T1: 0.7%).^1 7^ Our benign surgical resection rate was low at 2.5%, 10-fold lower than NLST and NELSON.^1 7^ The pathological confirmation rate and surgical resection rate are lower than reported in other trials. The exact reason for this is unclear but may be a consequence of higher deprivation and increased comorbidity in our population.

When reviewed retrospectively, five cancers diagnosed in the second screening round were present on baseline CT, and all were sub-5 mm solid nodules and therefore appropriately classified as negative in accordance with BTS guidelines.^4^ In all five cases, the cancers were stage I when detected, although with VDTs ranging from 51 to 163 days, there may have been a stage shift if we had adopted biennial rather than annual screening. This was also true for cancers that developed in individuals with true negative baseline scans; the estimated mean VDT of 49 days in this cohort suggests a more aggressive phenotype.

It is noteworthy that the proportion of attendees classified as false positive was three times lower in the second round than the first; the 3-month surveillance imaging rate was also 30% lower. This may be a consequence of having the baseline CT as a comparator; a similar finding was reported by the ITALUNG study investigators and suggests that the risk of screen-related harm may be greatest in the first round.^8^ Over both screening rounds, the false-positive rate was higher than NELSON but lower than other studies.^1 8–10^ In terms of baseline (T0) screening performance, the service had a sensitivity of 89.4% and specificity 97.1%. This represents a slightly lower sensitivity (93.8%) than NLST but a much improved specificity (73.4%).^1^

In conclusion, we have demonstrated that a targeted community-based lung cancer screening programme, delivered within the NHS, can engage those most at risk and detect a high proportion of curable early stage lung cancers.
